# Sarcoidose et spondylarthrite ankylosante: une association rare

**DOI:** 10.4314/pamj.v8i1.71080

**Published:** 2011-03-14

**Authors:** Fatima Zahra El Ouazzani, Latifa Tahiri, Nessrine Akasbi, Nadira Kadi, Taoufik Harzy

**Affiliations:** 1 Service de Rhumatologie, CHU Hassan II, Fès, Maroc

**Keywords:** Sarcoïdose, spondylarthrite ankylosante, spondylarthropathie, sacro-iliite, HLA B27

## Abstract

L’association spondylarthrite ankylosante et sarcoïdose est rare, seize cas ont été publiés dans la littérature, nous en rapportons un nouveau cas. Cette association qui pose des problèmes diagnostiques est probablement fortuite du fait de l’absence de facteurs génétiques prédisposant partagés et du petit nombre des cas publiés, mais elle suggère l’hypothèse d’une liaison physiopathologique entre eux.

## Introduction

La sarcoïdose est une granulomatose systémique de cause inconnue, dont les manifestations ostéoarticulaires sont polymorphes [1]. L’association sarcoïdose et spondylarthrite ankylosante (SA) est rare. Une revue de la littérature en dénombre 16 cas. À travers ce travail, nous en rapportons un nouveau cas.

## Patient et observation

Mme LS, âgée de 45ans, asthmatique depuis 10 ans contrôlée sous traitement, suivie depuis un an pour sarcoïdose ophtalmique cutanée et médiastinale confirmée par biopsie cutanée et traitée par corticoïdes avec bonne amélioration, ayant un frère suivi pour SA, qui présente depuis 7 ans des lombalgies, fessalgies à bascule, et douleurs enthésiques de type inflammatoire associés à une polyarthralgie des grosses articulations d’horaire inflammatoire.

L’examen clinique a trouvé une patiente en bon état général, avec douleur des épaules, sans synovite ni raideur. Le bilan biologique a objectivé une vitesse de sédimentation à 85 mm à la 1ère heure, C réactive protéine à 6 mg/l, le typage HLA B27 était positif.

**Figure 1:**
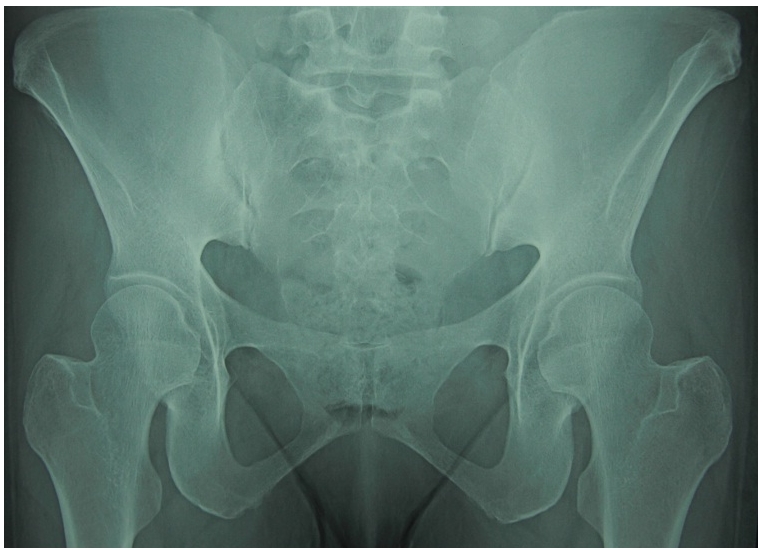
Radiographie du Bassin montant une Sacroilite stade III de Forestier bilatéra;e chez un patient marocain de 45 ans, atteint de Sarcoidose et spondylarthrite ankylosante.

**Figure 2:**
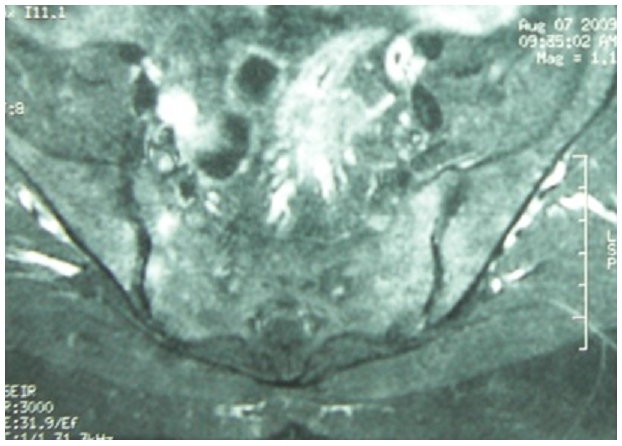
IRM des Sacro-iliaques montrant un œdème osseux sous chondral témoignant d’une Sacroilite bilatérale bilatérale chez un patient marocain de 45 ans, atteint de Sarcoidose et spondylathrite ankylosante

La radiographie du bassin a montré une Sacroiliite bilatérale stade III de Forestier (Figure 1), confirmée par l’IRM du bassin (Figure 2), la radiographie des arrières pieds a objectivé un blindage calcanéen bilatéral.

Le diagnostic de SA forme axiale périphérique et enthésique associée à la sarcoïdose a été retenu devant : la réponse aux critères d’Amor : 12 points, critères de New York, et de l’European Spondyloarthropathy Study Group (ESSG); 1 critère majeur et 2 mineurs. Le Bath Ankylosing Spondylitis Disease Activity Index (BASDAI) était à 3 et le Bath Ankylosing Spondylitis Functional Index (BASFI) à 1.8. La patiente a bénéficié d’un traitement par Célécoxib et de la rééducation avec bonne amélioration.

## Discussion

L’association sarcoïdose et spondylarthrite ankylosante soulève des questions sur le lien entre ces deux affections et pose parfois des problèmes d’ordre diagnostique.

La sarcoïdose est une granulomatose systémique de cause inconnue, dont les manifestations ostéoarticulaires sont polymorphes. L’atteinte articulaire chronique est très rare et a été rapportée à 0,2 %, intéressant principalement les patients noirs de sexe masculin. De même que l’atteinte du bassin qui est rare [1]. La sarcoïdose en causant une sacro-iliite peut mimer une spondylarthropathie [2]. Cette atteinte a été longuement controversée. Selon Kremer et al [3], cette atteinte peut avoir trois origines différentes : une manifestation rhumatologique originale de la sarcoïdose; une atteinte infectieuse notamment tuberculeuse; ou une sacro-iliite inflammatoire dans le cadre d’une association à une authentique spondylarthrite ankylosante. Dans une étude prospective ayant porté sur 61 patients atteints de sarcoïdose, Erb et al.[2] ont trouvé une prévalence très anormale de SA de 6,6 %. Toutefois, si quatre patients avaient bien une sacro-iliite radiologique, un seul d’entre eux avait une sacro-iliite bilatérale et était porteur de l’antigène HLA B27. Les trois autres ne souffraient que d’une sacro-iliite unilatérale et étaient HLA B27 négatifs.

Seize cas associant une sarcoïdose à une spondylarthropathie ont été publiés dans la littérature [3-8]. Le diagnostic de la spondylarthropathie a précédé celui de la sarcoïdose dans 5 cas et la survenue concomitante des deux affections a été observée dans 5 autres. Il faut noter que bien qu’il ait été annoncé que HLA-B27 pourrait avoir un rôle dans le développement de sacroiliite [2], seulement 3 de 15 patients ayant l’association sarcoïdose-spondyloarthropathie ont révélé la positivité HLA-B27 [4] et notre cas est le quatrième. Dans notre observation la sarcoïdose était connue et le diagnostic était posé sur des arguments cliniques et histologiques; Celui de la SA était évident devant le terrain génétique, les critères cliniques, radiologiques notamment en IRM et thérapeutiques répondant ainsi aux critères diagnostiques de la SA.

Ce sont deux entités distinctes et leur association est probablement fortuite du fait de: l’absence de facteurs génétiques prédisposant partagés et du petit nombre de cas publiés mais: cela suggère l’hypothèse de mécanismes physiopathologiques communs qui restent à définir.

## Conclusion

Le cadre nosologique des spondylarthropathies et leurs mécanismes physiopathologiques sont régulièrement discutés. Un intérêt particulier doit être porté aux formes frontières de cette pathologie, ainsi que ses intrications avec la sarcoïdose, dont les traitements et la physiopathologie sont mieux connus. La prise en charge des patients associant spondylarthropathie et sarcoïdose n’est pas définie. Le dépistage, au cours des spondylarthropathies, de points d’appels cliniques doit être répété et peut modifier leur suivi et leur traitement.

## Conflit d’intérêts

Les auteurs ne déclarent aucun conflit d’intérêt.

## Contribution des auteurs

Tous les auteurs ont contribué à la réalisation ce travail selon les critères de l’ICMJE.
